# Barriers to Communication in Undergraduate Medical Education: Perspectives of Students From Karnataka, India

**DOI:** 10.7759/cureus.70364

**Published:** 2024-09-28

**Authors:** Nandakishsore Bommeri, Imaad Mohammed Ismail, Kahkashan Azeez

**Affiliations:** 1 Respiratory Medicine, Adichunchanagiri Institute of Medical Sciences, Mandya, IND; 2 Community Medicine, Yenepoya Medical College, Mangaluru, IND; 3 Physiology, Yenepoya Medical College, Mangaluru, IND

**Keywords:** communication barriers, communication challenges, communication solutions, environmental barriers, language barriers, medical education, psychological barriers, teacher-related issues

## Abstract

Background

Communication barriers in medical education can impede learning. Understanding these barriers from students' perspectives is crucial for developing effective solutions. The objectives of this study were to identify the barriers to communication in undergraduate medical education and their solutions from students' perspectives as well as to determine the demographic factors associated with these barriers.

Materials and methods

A cross-sectional study was conducted from May 2018 to July 2019 among undergraduate students at a medical college in Karnataka. Using a stratified sampling method, data was collected from 400 students via a pretested semi-structured questionnaire. The data was analyzed using descriptive statistics, and logistic regression analysis was performed to determine the association between demographic factors and barriers to communication.

Results

Students identified several communication barriers, primarily in the domains of language, environment, psychology, and teacher-related issues. Key barriers in theory classes included the use of medical jargon, overcrowding, faulty microphones, absent-mindedness, peer pressure, fast-paced teaching, monotonous sessions, and information overload. In practical classes, barriers were overcrowding, absent-mindedness, and fast-paced teaching, and for clinical case discussions, barriers included difficulty understanding the local language, overcrowding, and leg pain. Proposed solutions by students for theory classes included simpler explanations, smaller batches, microphone maintenance, interactive lectures, and breaks between classes. For practical classes, solutions were smaller practical batches, interactive sessions, and simpler explanations of topics, while for case discussion, solutions included optional local language classes, smaller groups, and better seating arrangements.

Conclusion

This study highlights key communication barriers in medical education and underscores the influence of demographic factors such as gender and academic year. The solutions proposed by the students are practical and could enhance communication and the overall educational experience if implemented in medical colleges across India.

## Introduction

Communication is the science and practice of transmitting the information. It is defined as "a process by which information is exchanged between individuals through a common system of symbols, signs, or behaviour" [1]. All communities largely depend on their communication systems. Even the people of the ancient world realized that good communication was the secret to a successful civilization and put great effort into improving their communication capabilities. In today's era of information revolution, the significance of communication has become even more pronounced.

The communication process comprises five fundamental components: the sender, receiver, message, channel of communication, and feedback [2]. Any disruption in this process can result in inefficiencies or misinterpretations, ultimately undermining its effectiveness. Such miscommunications may manifest in various ways, including heightened stress levels, increased irritation, strained interpersonal relationships, decreased productivity across industries, and hindered learning processes. Barriers to communication refer to any obstacle or impediment that hinders the effective exchange of information between individuals or groups. Various barriers to communication, including linguistic, cultural, physical, and environmental, have been observed in different settings, such as workplaces and educational institutions [3-7].

In classroom communications, the teacher serves as the primary source of information, responsible for selecting and organizing content for the students, who act as receivers. Information is conveyed through various channels, including speech, demonstrations, or PowerPoint presentations. The students in a medical college comprise a diverse group, hailing from different geographic areas of the country and exhibiting varying socioeconomic statuses, customs, beliefs, and languages. It has been observed that students often encounter difficulties in hearing or understanding the explanations provided by medical teachers [8-10]. This could be attributed to various factors such as a noisy environment, microphone issues, language barriers, differences in perception, and classroom distractions [11,12]. Ineffective communication can lead to poor subject learning, inadequate clinical skills development, diminished overall academic performance, and mental frustration among students [13].

Communication barriers in undergraduate teaching are an often-discussed topic by the medical fraternity, yet there is a lack of concrete research in this area. There are gaps in our understanding of the communication barriers that exist in undergraduate medical teaching. Identifying these gaps from students' perspectives and rectifying them will enhance teaching and learning experiences. Thus, this study was undertaken with the objectives of identifying barriers to communication in undergraduate medical education and their solutions from students' perspectives as well as determining the demographic factors associated with these barriers at a medical college in Karnataka, India.

## Materials and methods

This cross-sectional study was conducted from May 2018 to July 2019 at Yenepoya Medical College, a private institution affiliated with Yenepoya (Deemed to be University) in Mangalore, Karnataka, India. The study population comprised 600 undergraduate students from the first to final years. Ethical clearance was obtained from the Institutional Ethics Committee. A sample size of 400 was determined using the formula n=z^2^pq/d^2^, with a prevalence (p) of 0.5 chosen due to the novelty of the study and absolute precision (d) of 5%. A stratified random sampling method was used to ensure representation from different academic years. A list of all the students with their roll numbers was obtained from the principal's office. There were approximately 150 medical students each in the first, second, third, and final years. Each year was considered as a stratum, and 25% of the total required sample, i.e., 100 students, were selected from each year using computer-generated random numbers. If a specific student was unavailable or unwilling to participate during the data collection period, the subsequent student in the roll number sequence was invited to take part in the study. Thus, a total of 400 medical students were selected for the study.

The authors developed a semi-structured questionnaire (see Appendices) based on a review of existing literature and expert consultations. It was validated by three subject experts and pilot-tested with a small group of students from a neighboring medical college to refine questions for clarity and relevance. The questionnaire had two parts: the first part focused on socio-demographic variables such as age, gender, medical batch, and residence. The second part included questions on various communication barriers and their solutions, categorized into language, environmental, psychological, social, teacher-related issues, physiological, and others. Information on barriers in theory, practical, and clinical case discussions was collected separately.

The data were collected by the primary investigator during lunch breaks, after class hours, or if any other opportunity arose such as a class being canceled. The students selected by the sampling method were approached individually and were explained about the study. If they were willing to participate, written informed consent was obtained, and they were provided with a printed copy of the study questionnaire to fill out and return. The questionnaire was anonymous, and the confidentiality of the participants was maintained throughout the study. The collected data was entered into Microsoft Office Excel (version Office 2019, Microsoft Corporation, Redmond, Washington, United States) and analyzed on IBM SPSS Statistics for Windows, Version 25.0 (Released 2017; IBM Corp., Armonk, New York, United States). Descriptive statistics were used to summarize the data, including frequencies and percentages of identified barriers. Logistic regression analysis was performed to assess demographic factors associated with barriers to communication. The variables with a p-value of less than 0.25 in the univariate analysis were included in the multivariate analysis, and a p-value of less than 0.05 was considered statistically significant.

## Results

**Figure 1:**
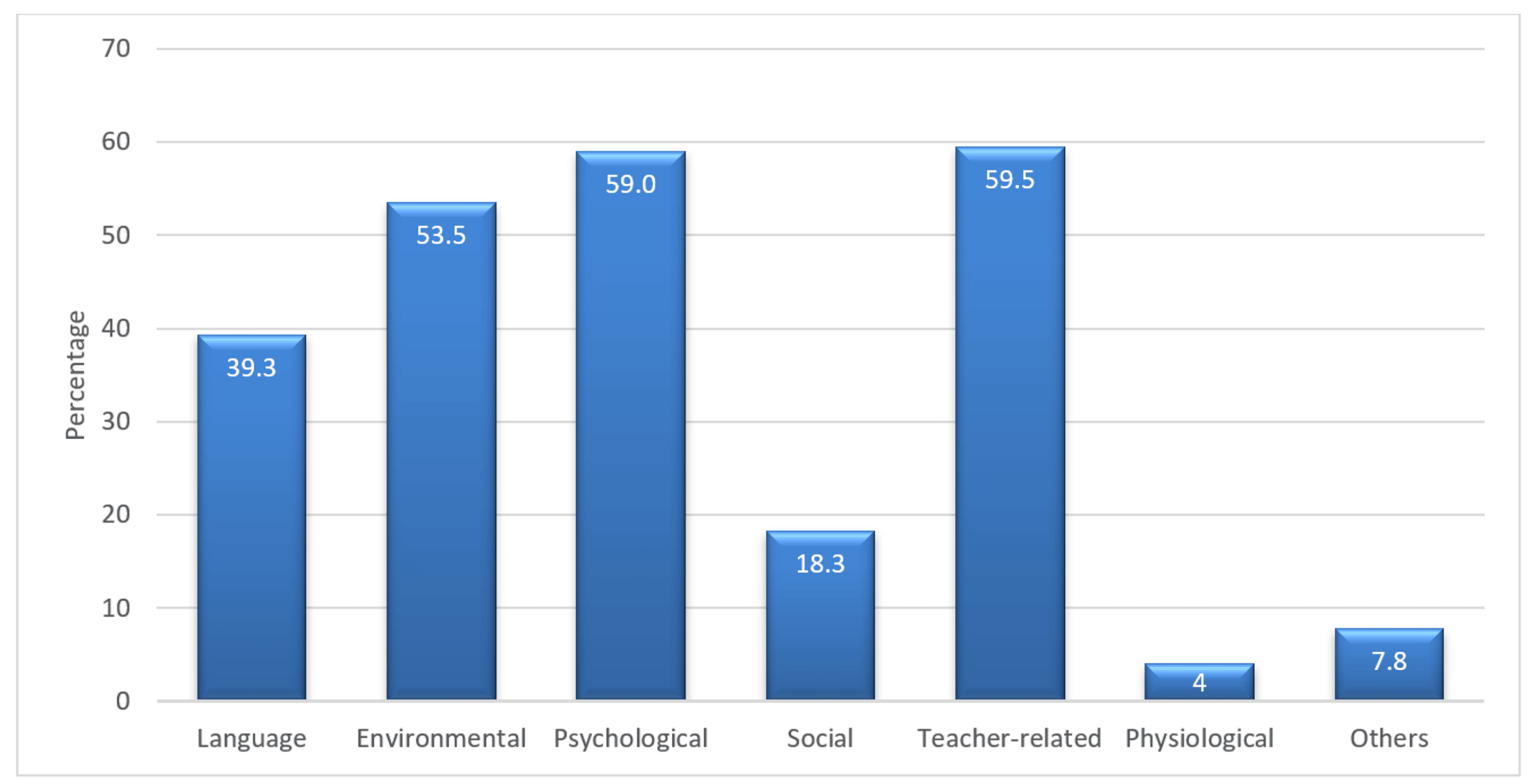
Bar chart showing communication barrier domains in undergraduate medical education, expressed as a percentage

A total of 400 medical students participated in the study, with an average age of 20.9 years (±1.5). Among the participants, 46.7% were males, while the remaining were females. The majority of students (86%) were from urban backgrounds. Language, environmental, psychological, and teacher-related domains were reported as the most important domains affecting communication in undergraduate medical education (Figure 1).

In the language domain, the use of medical jargon and local language barriers were most commonly reported, with students suggesting clearer explanations and local language training as solutions (Table 1). Environmental barriers included overcrowding and poor microphone quality, with proposed solutions such as smaller class groups and improved microphone maintenance.

**Table 1:** Language and environmental communication barriers and their solutions as reported by the students (N=400)

Domain	Theory class		Practical class		Clinical case	
	N	%	N	%	N	%
Language barriers						
Accent	37	9.3	14	3.5	13	3.3
Medical jargons	56	14	27	6.8	38	9.5
Local language	19	4.8	18	4.5	46	11.5
Solutions to language barriers						
Speak slowly with repetition	17	4.3	9	2.3	6	1.5
Clear explanation	34	8.5	11	2.8	16	4
Training of teachers	6	1.5	3	0.8	4	1
Local language training classes	14	3.5	17	4.3	30	7.5
Environmental barriers						
Overcrowding	92	23	45	11.3	59	14.8
Small font size of PowerPoint	15	3.8	1	0.3	0	0
Poor microphone quality	71	17.8	31	7.8	10	2.5
Inadequate ventilation: fans	3	0.8	17	4.3	4	1
Poor lighting	27	6.8	2	0.5	1	0.3
Solutions to environmental barriers						
Small groups	33	8.3	20	5	26	6.5
Appropriate font size for PowerPoint	6	1.5	0	0	0	0
Microphone maintenance	51	12.8	22	5.5	6	1.5
Fans' installation	0	0	6	1.5	1	0.3

In the psychological domain, the students perceived absent-mindedness, batchmate disturbances, and difficulty in understanding the subject as the main barriers and proposed more interactive teaching sessions as its solution (Table 2). Social barriers were primarily related to peer pressure, with students suggesting the need for greater open-mindedness among peers.

**Table 2:** Psychological and social communication barriers and their solutions as reported by the students (N=400)

Domain	Theory class		Practical class		Clinical case	
	N	%	N	%	N	%
Psychological barriers						
Absent-mindedness	165	41.3	66	16.5	41	10.3
Mobile phone distractions	33	8.3	12	3	8	2
Batchmate disturbances	46	11.5	21	5.3	14	3.5
Difficulty in understanding the subject	46	11.5	19	4.8	13	3.3
Targeted questioning of certain students	10	2.5	6	1.5	5	1.3
Lack of interest in class	14	3.5	5	1.3	7	1.8
Solutions to psychological barriers						
More interactive sessions	92	23	23	5.8	12	3
Make sessions interesting	15	3.8	9	2.3	4	1
Detailed explanations	8	2	0	0	1	0.3
Avoid targeted questioning	1	0.3	1	0.3	1	0.3
Social barriers						
Peer pressure	59	14.8	26	6.5	13	3.3
Perception difference with teacher	13	3.3	4	1	4	1
Solutions to social barriers						
Open-mindedness by peers	5	1.3	2	0.5	2	0.5

Teacher-related barriers included fast-paced teaching, information overload, and monotonous teaching styles. The suggested solutions included providing simpler explanations (Table 3). Physiological barriers were less frequently reported but included issues like low vision and leg pain, with seating arrangements proposed as a solution for leg pain. Continuous classes and lack of orientation were reported under other barriers, with breaks between classes suggested as a solution.

**Table 3:** Teacher-related, physiological, and other communication barriers and their solutions as reported by the students (N=400)

Domain	Theory class		Practical class		Clinical case	
	N	%	N	%	N	%
Teacher-related barriers						
Fast-paced teaching	126	31.5	40	10	17	4.3
Information overload	113	28.3	31	7.8	31	7.8
Monotonous teaching styles	49	12.3	1	0.3	8	2
Strictness	5	1.3	1	0.3	1	0.3
Solutions to teacher-related barriers						
Easy explanation of the topic	85	21.3	12	3	7	1.8
Physiological and other barriers						
Low vision	10	2.5	0	0	0	0
Leg pain	0	0	0	0	7	1.8
Early morning classes (8:00 a.m.)	5	1.3	0	0	0	0
Continuous classes	14	3.5	2	0.5	1	0.3
Lack of class orientation	16	4	3	0.8	0	0
Solutions to physiological and other barriers						
Seating arrangement	0	0	0	0	3	0.8
Commence classes at 9:00 a.m.	5	1.3	0	0	0	0
Breaks between classes	13	3.3	3	0.8	1	0.3

In theory classes, the major problems identified included the accent, use of medical jargon, overcrowding, faulty microphones, absent-mindedness, mobile phone use, distractions by batchmates, difficulty in understanding the subject, peer pressure, fast-paced teaching, monotonous sessions, and information overload. In practical classes, the main issues were overcrowding, absent-mindedness, and fast-paced teaching. During clinical case discussions, the primary problems were the inability to understand the local language, overcrowding, leg pain due to standing, and information overload by the teacher.

Solutions for improving theory classes, as perceived by students, included providing simpler explanations of topics, speaking slowly with repetition, creating smaller batches, maintaining microphones properly, making lectures more interactive, and incorporating breaks between classes. Solutions for improving practical classes included smaller practical batches, more interactive sessions, and simpler explanations of topics. For clinical case discussions, the suggested solutions included offering local language training classes, forming smaller groups for case discussions, and providing seating arrangements.

The study identified selected associations between demographic variables such as gender, residence, and medical batch with various domains of barriers to communication. Language barriers were reported significantly more by female students and those in their third year of medical studies (Table 4). Similarly, environmental barriers were also significantly higher among females and third-year students (Table 5).

**Table 4:** Association of demographic variables with language barriers in undergraduate medical education (N=400)

Variable	Total	Language barriers reported	Univariate analysis		Multivariate analysis	
	N	n (%)	Unadjusted OR (95% CI)	P-value	Adjusted OR (95% CI)	P-value
	400	157 (39.3)				
Gender						
Male	187	62 (33.2)	0.62 (0.41-0.93)	0.019	0.58 (0.39-0.89)	0.012
Female	213	95 (44.6)	Reference		Reference	
Residence						
Urban	344	133 (38.7)	0.84 (0.47-1.49)	0.551	-	-
Rural	56	24 (42.9)	Reference		-	-
Batch						
First year	100	32 (32)	1.05 (0.56-1.90)	0.879	0.99 (0.55-1.83)	0.999
Second year	100	44 (44)	1.75 (0.98-3.12)	0.059	1.77 (0.98-3.12)	0.057
Third year	100	50 (50)	2.23 (1.25-3.97)	0.007	2.26 (1.26-4.04)	0.006
Fourth year	100	31 (31)	Reference		Reference	

**Table 5:** Association of demographic variables with environmental barriers in undergraduate medical education (N=400)

Variable	Total	Environmental barriers reported	Univariate analysis		Multivariate analysis	
	N	n (%)	Unadjusted OR (95% CI)	P-value	Adjusted OR (95% CI)	P-value
	400	214 (53.5)				
Gender						
Male	187	86 (46)	0.57 (0.38-0.84)	0.005	0.51 (0.34-0.77)	0.001
Female	213	126 (60.1)	Reference		Reference	
Residence						
Urban	344	184 (53.5)	0.99 (0.57-1.76)	0.991	-	-
Rural	56	30 (53.6)	Reference		-	-
Batch						
First year	100	38 (38)	0.64 (0.36-1.12)	0.117	0.59 (0.33-1.05)	0.074
Second year	100	59 (59)	1.49 (0.86-2.62)	0.157	1.56 (0.86-2.67)	0.151
Third year	100	68 (68)	2.21 (1.25-3.93)	0.007	2.26 (1.26-4.05)	0.006
Fourth year	100	49 (49)	Reference		Reference	

Psychological barriers were more prevalent among females and tended to increase from the first to the second years of medical college, with a lower occurrence reported in the first year. Although psychological barriers were more frequently reported by students from rural backgrounds, this association was not statistically significant in multivariate logistic regression (Table 6). The social barriers were perceived to be higher by third-year medical students, and no association was noted with gender and residence (Table 7).

**Table 6:** Association of demographic variables with psychological barriers in undergraduate medical education (N=400)

Variable	Total	Psychological barriers reported	Univariate analysis		Multivariate analysis	
	N	n (%)	Unadjusted OR (95% CI)	P-value	Adjusted OR (95% CI)	P-value
	400	236 (59)				
Gender						
Male	187	91 (48.7)	0.45 (0.30-0.67)	<0.001	0.38 (0.25-0.59)	<0.001
Female	213	145 (68.1)	Reference		Reference	
Residence						
Urban	344	196 (57)	0.53 (0.29-0.98)	0.041	0.58 (0.30-1.12)	0.105
Rural	56	40 (71.4)	Reference		Reference	
Batch						
First year	100	39 (39)	0.50 (0.29-0.88)	0.017	0.44 (0.25-0.79)	0.006
Second year	100	72 (72)	2.02 (1.12-3.64)	0.019	2.07 (1.13-3.81)	0.019
Third year	100	69 (69)	1.75 (0.98-3.12)	0.059	1.74 (0.95-3.17)	0.071
Fourth year	100	56 (56)	Reference		Reference	

**Table 7:** Association of demographic variables with social barriers in undergraduate medical education (N=400) Since only the variable "Batch" qualified for multivariate analysis, the results are identical to those obtained in the univariate analysis.

Variable	Total	Social barriers reported	Univariate analysis		Multivariate analysis	
	N	n (%)	Unadjusted OR (95% CI)	P-value	Adjusted OR (95% CI)	P-value
	400	73 (18.3)				
Gender						
Male	187	34 (18.2)	0.99 (0.57-1.65)	0.974	-	-
Female	213	39 (18.3)	Reference		-	-
Residence						
Urban	344	61 (17.7)	0.79 (0.39-1.59)	0.507	-	-
Rural	56	12 (21.4)	Reference		-	-
Batch						
First year	100	11 (11)	0.76 (0.33-1.77)	0.522	0.76 (0.33-1.77)	0.522
Second year	100	13 (13)	0.92 (0.41-2.07)	0.836	0.92 (0.41-2.07)	0.836
Third year	100	35 (35)	3.31 (1.65-6.65)	0.001	3.31 (1.65-6.65)	0.001
Fourth year	100	14 (14)	Reference		Reference	

Teacher-related barriers were significantly higher among female students, with these barriers being less common in the first year but increasing in the second and third years (Table 8). No significant association was found between demographic variables and physiological barriers (Table 9). Additionally, the number of students reporting other barriers was too low to allow for meaningful interpretation (Table 10).

**Table 8:** Association of demographic variables with teacher-related barriers in undergraduate medical education (N=400)

Variable	Total	Teacher-related barriers reported	Univariate analysis		Multivariate analysis	
	N	n (%)	Unadjusted OR (95% CI)	P-value	Adjusted OR (95% CI)	P-value
	400	238 (59.5)				
Gender						
Male	187	97 (51.9)	0.55 (0.37-0.82)	0.004	0.46 (0.29-0.71)	0.001
Female	213	141 (66.2)	Reference		Reference	
Residence						
Urban	344	200 (58.1)	0.66 (0.36-1.20)	0.170	0.75 (0.39-1.43)	0.380
Rural	56	38 (67.9)	Reference		Reference	
Batch						
First year	100	37 (37)	0.56 (0.32-0.99)	0.047	0.52 (0.29-0.92)	0.025
Second year	100	80 (80)	3.84 (2.05-7.19)	<0.001	3.99 (2.11-7.59)	<0.001
Third year	100	70 (70)	2.24 (1.26-4.01)	0.006	2.27 (1.25-4.10)	0.007
Fourth year	100	51 (51)	Reference		Reference	

**Table 9:** Association of demographic variables with physiological barriers in undergraduate medical education (N=400)

Variable	Total	Physiological barriers reported	Univariate analysis		Multivariate analysis	
	N	n (%)	Unadjusted OR (95% CI)	P-value	Adjusted OR (95% CI)	P-value
	400	16 (4)				
Gender						
Male	187	7 (3.7)	0.88 (0.32-2.42)	0.806	-	-
Female	213	9 (4.2)	Reference		-	-
Residence						
Urban	344	15 (4.4)	2.51 (0.33-19.37)	0.362	-	-
Rural	56	1 (1.8)	Reference		-	-
Batch						
First year	100	4 (4)	4.13 (0.45-37.57)	0.209	-	-
Second year	100	4 (4)	4.13 (0.45-37.57)	0.209	-	-
Third year	100	7 (7)	7.45 (0.90-61.73)	0.063	-	-
Fourth year	100	1 (1)	Reference		-	-

**Table 10:** Association of demographic variables with other barriers in undergraduate medical education (N=400) Since only the variable "Batch" qualified for multivariate analysis, the results are identical to those obtained in the univariate analysis.

Variable	Total	Other barriers reported	Univariate analysis		Multivariate analysis	
	N	n (%)	Unadjusted OR (95% CI)	P-value	Adjusted OR (95% CI)	P-value
	400	31 (7.8)				
Gender						
Male	187	12 (6.4)	0.70 (0.33-1.48)	0.350	-	-
Female	213	19 (8.9)	Reference		-	-
Residence						
Urban	344	27 (7.8)	1.11 (0.37-3.29)	0.855	-	-
Rural	56	04 (7.1)	Reference		-	-
Batch						
First year	100	12 (12)	13.50 (1.72-105.9)	0.013	13.50 (1.72-105.9)	0.013
Second year	100	12 (12)	13.50 (1.72-105.9)	0.013	13.50 (1.72-105.9)	0.013
Third year	100	6 (6)	6.32 (0.75-53.48)	0.091	6.32 (0.75-53.48)	0.091
Fourth year	100	1 (1)	Reference		Reference	

## Discussion

Effective communication between teachers and students is crucial for fostering optimal learning environments. This study was undertaken to understand the different barriers to communication that exist in undergraduate medical education and their solutions from students' perspectives as well as to examine the demographic factors influencing these barriers. The students reported various barriers to communication predominantly under the domains of language, environmental, psychological, and teacher-related. The extent of the barriers varied across theoretical, practical, and clinical case discussion settings, and similarly, the solutions varied accordingly.

The present study had 400 medical students equally spread across all the medical years. A higher proportion of female medical students (53.3%) was observed compared to male medical students (46.7%), a common pattern in most medical colleges of the region, likely due to the higher female literacy rate and sex ratio [14]. The study had a significant majority of students from urban areas, possibly due to better educational opportunities and higher financial status to afford medical college expenses.

Language domain

A language barrier was reported by 39.3% of students. The findings align with a study by Gul et al. at Pakistan Medical College, where almost 50% of students encountered language issues, and a study by Abass et al. at Egypt Medical College, in which 56.6% of students faced language problems [7,8]. In the current study, accent issues and the use of medical jargon without proper explanation were identified as significant problems, primarily in theory classes but also in practical and clinical classes. Accent variations often arise due to regional and urban-rural disparities in English pronunciation. When it comes to medical jargon, teachers need to recognize that despite students' backgrounds in science, many medical terms may still be unfamiliar to them. Therefore, medical terminology should be gradually introduced to students using simpler language.

Students attending medical colleges hail from diverse backgrounds, including local students from the same state, non-local students from other states, and students admitted under the non-resident Indian category. Since the introduction of the National Eligibility cum Entrance Test for Undergraduates (NEET-UG) in 2013, which serves as a unified entrance examination for MBBS admissions across India, it has been observed that more students are crossing state borders for medical education [15]. Consequently, some students encounter difficulties in comprehending the local language, particularly during clinical case discussions in their second and third years. As a solution, students have recommended the introduction of optional local language classes.

Female students reported language barriers more than their male counterparts, and this may be partly attributed to the fact that males generally have more outdoor interactions and may have a better understanding of local languages and accents. The third-year students reported significantly higher language barriers, which can be attributed to their clinical case discussions as explained earlier. Similar observations were made in a study by Sheikh et al. in Saudi Arabia where non-native medical students identified language barriers as a significant obstacle to effective clinical learning and recommended advanced occupation-related language courses to address this issue [16].

Environmental domain

The interaction between teachers and students is not only about their comprehension but also about the study environment, including factors such as class size, lighting, ventilation, and the use of audio-visual aids [11]. It is particularly challenging for a teacher to effectively engage with 150 or more students in a theory class, and 23% of students reported difficulty in following the class due to overcrowding. Similarly, in clinical case discussions in hospital wards, it's nearly impossible for a student to understand a case if they are not in a position to even see patients. It was found that one in every seven students was facing this problem. These findings are similar to those of Pal et al., where 58% of teachers felt a large class to be a barrier [4]. Faulty microphones also emerged as a major issue, likely due to their frequent use by multiple people. Implementing smaller batches and ensuring regular microphone maintenance could help mitigate these problems.

Environmental barriers were reported significantly more often by female students. This may be due to overcrowding, particularly during clinical case discussions, where female students might find it challenging to find a good position among their male peers in order to view the patient and teacher. Additionally, the generally shorter height of female students compared to males might hinder their ability to clearly see the patient. Third-year students also reported higher environmental barriers compared to other batches, which is possibly due to the faulty microphone in the lecture hall commonly used by third-year students.

Psychological domain

One of the biggest barriers reported by students was absent-mindedness. The ability of students to focus their attention during class is a crucial factor affecting learning. Attention is influenced by psychological, rhetorical, and linguistic aspects of communication in the classroom [17]. In the present study, it was found that four out of 10 students were unable to concentrate for an hour-long theory class. A similar study conducted in a medical college in Abbottabad by Mustafa et al. reported that 92% of students were unable to concentrate for more than 30 minutes [18]. Although the use of mobile phones is prohibited during class hours, some students reported using them or witnessing their peers using them, and they felt that mobile phones were significant distractors during theory lectures. A study by Shrivastava and Shrivastava found that over 83% of teachers considered mobile phones to be significant distractors and over 50% of students were found to be using mobile phones in the classroom [19].

Approximately 12% of students reported having difficulty understanding the subject because they found it too challenging. This phenomenon is similar in most professional colleges, where slow learners find it difficult to cope with the subjects. Some students also mentioned that they were not interested in pursuing a career in the medical field and only took up the seats because it was recommended by their families. Students felt that they could concentrate better in class if the sessions were more interactive, a suggestion also recommended in studies by Begum et al. and Mehta and Bhandari [20,21]. Buch et al. proposed interactive methods like the use of multiple-choice questions, teamwork, brainstorming, and confusion techniques to hold students' attention [22].

Psychological barriers were perceived more by the female students, possibly due to batchmate disturbance and potentially better reporting by the female students. Students from rural areas reported higher psychological barriers, which could be attributed to difficulty in understanding the subject, although this was not statistically significant in multivariate analysis. Second-year students reported significantly higher psychological barriers compared to first-year students, which may be due to absent-mindedness, possibly stemming from a sense of seniority and a more relaxed attitude compared to their first-year peers.

Social domain

The impact of peer pressure in classrooms is often underestimated. In the present study, 14.8% of students reported difficulties in effective communication as a result of peer pressure. Students often avoid participating in class discussions or asking questions to the teacher due to the fear of being judged by their peers or to conform to a group that typically remains silent. This behavior can also result from a lack of confidence and differences in cultural backgrounds. Therefore, it is essential to develop cultural competence in medical colleges and foster an environment that promotes healthy learning with mutual respect [23,24].

The study also found that social barriers were significantly more prevalent among third-year medical students. This may be attributed to specific internal dynamics within the batch, as other factors, such as admission criteria and teaching-learning methods, remain consistent with those of other batches.

Teacher-related domain

Many students have reported that they were struggling to keep up with the theory class because the teacher was covering the topics too quickly, leading to information overload. Given the extensive nature of the medical curriculum and the impracticality of covering every topic in detail, focusing on the key concepts would enhance students' comprehension of the topic while also facilitating self-directed study. Furthermore, monotonous teaching has been cited as a reason for students' lack of interest in the class, which could be improved by incorporating voice modulation, varying teaching methods, and making the teaching more interactive. Interestingly, unlike a previous study by Banfield et al., the strictness of teachers was not identified as an important communication barrier in this study [25].

Teacher-related barriers were perceived more frequently by female students. Since the teaching methods and teachers are the same for both male and female students, this difference is challenging to explain and may be attributed to better reporting of these barriers by female students. These barriers were reported less frequently by first-year students but significantly more by second- and third-year students. In the first year, students have only three main subjects and often develop close relationships with their teachers. As students progress to the second and third years, they are introduced to a greater number of subjects taught by different teachers, which can make it challenging for them to cope with the teaching pace and information load. This aligns with observations by Westerman and Teunissen, who noted that as students transition into clinical years, the increased complexity of subjects and variability in teaching methods can overwhelm them, leading to greater difficulty in adapting to teaching styles and maintaining pace with the curriculum [26].

Physiological domain

Physiological barriers did not significantly affect classroom communication in this study. Only 2.5% of students have visual impairments, which can be addressed by adjusting their seating position and providing corrective eyewear. In medical colleges, students often have three-hour clinical case discussions, which require them to stand. This has resulted in some students experiencing leg pain and cramps, leading to a decreased ability to focus in class. Providing seating arrangements and incorporating short breaks would address the issue. No association was found between demographic variables and physiological barriers.

Other domain

The other barrier reported was related to the scheduling of classes. Some students find it inconvenient to attend early morning classes at 8:00 a.m. because they often skip breakfast due to lack of time and end up feeling hungry during class. Additionally, back-to-back classes make them feel exhausted, affecting their active class participation. As a solution, students have suggested to have breaks every two to three hours. The statistical analysis indicates that these barriers were more prevalent among first- and second-year students. However, it is difficult to draw a meaningful interpretation due to the low frequencies.

The strength of the study lies in the uniqueness of examining barriers to communication in medical education from the student's viewpoint, as most studies have considered only the teachers' perspective. The study does have a few limitations. First, since the study was conducted at a single institution, its findings may have limited generalizability to other medical colleges. Additionally, the study was conducted in a medical college affiliated with a private deemed university, which generally offers better facilities than those at non-deemed university colleges, potentially resulting in a lower number of reported barriers compared to these institutions. Furthermore, students may not have accurately reported all barriers and might have provided a limited number of solutions due to potential disinterest in completing the questionnaire.

## Conclusions

This study identified the communication barriers faced by undergraduate medical students, particularly in the domains of language, environment, psychology, and teacher-student issues. Students offered practical solutions, such as simplifying explanations, introducing optional local language classes, managing class sizes, improving microphone maintenance, and increasing the interactivity of sessions, all of which could be readily implemented in medical colleges across the country. These strategies have the potential to enhance communication and improve the overall educational experience. The study also highlighted that communication barriers were more frequently reported by female students and those in their second and third years, underscoring the need to consider demographic factors in addressing these issues. Further research from the perspectives of students across different medical institutions is recommended to strengthen and expand the current evidence base.

## Appendices

Questionnaire

Batch:

Serial number:

Age (in years):

Gender:

(1) Male

(2) Female

Residence:

(1) Urban

(2) Rural

In your MBBS classes, you may have faced problems with listening and understanding what the teacher is explaining to you. The following tables show many areas in which a communication barrier may arise between a teacher and a student. You are requested to kindly write in the appropriate boxes the problems which you have faced in your MBBS classes and the solutions to it.

**Table 11:** Language barriers and its solutions in undergraduate medical education

	Theory class	Practical class	Clinical case discussion
Language problems/barriers (e.g., language problem, accent problem, difficulty in understanding medical terms, etc.)			
Solutions for language barriers			

**Table 12:** Environmental barriers and its solutions in undergraduate medical education

	Theory class	Practical class	Clinical case discussion
Environmental barriers (e.g., mic, speaker, display projector, light problem, noisy surroundings, overcrowding, etc.)			
Solutions for environmental barriers			

**Table 13:** Psychological barriers and its solutions in undergraduate medical education

	Theory class	Practical class	Clinical case discussion
Psychological/mental barriers (e.g., emotional disturbance, absent-mindedness, subjects too difficult to understand, not interested in learning, distractors such as mobile phone, batchmate disturbance, etc.)			
Solutions for psychological/mental barriers			

**Table 14:** Social barriers and its solutions in undergraduate medical education

	Theory class	Practical class	Clinical case discussion
Social or cultural barriers (e.g., peer pressure, difference in socioeconomic status, culture, perceptions, etc.)			
Solutions for social or cultural barriers			

**Table 15:** Teacher-related barriers and its solutions in undergraduate medical education

	Theory class	Practical class	Clinical case discussion
Teacher-related barriers (e.g., too fast, too much information, teacher turning towards the board, etc.)			
Solutions for teacher-related barriers			

**Table 16:** Physiological barriers and its solutions in undergraduate medical education

	Theory class	Practical class	Clinical case discussion
Physiological barriers (problems with hearing and speech) (e.g., low vision, partial deafness, any disability)			
Solutions for physiological barriers			

**Table 17:** Other barriers and its solutions in undergraduate medical education

	Theory class	Practical class	Clinical case discussion
Other barriers (you can write any other problem faced by you)			
Solutions for other barriers			
